# Case Report: Pericardial patch repair of mitral annulus and mitral valve for a left atrial dissection caused by parasitic infective endocarditis

**DOI:** 10.3389/fcvm.2023.1239019

**Published:** 2023-11-28

**Authors:** PeiShan Chu, Yi Tang, XinPei Liu, Qi Miao

**Affiliations:** ^1^ Department of Cardiac Surgery, Peking Union Medical College Hospital, Dongcheng District, Beijing, China; ^2^ Chinese Academy of Medical Sciences & Peking Union Medical College, Beijing, China

**Keywords:** left atrial dissection, parasite, infectious endocarditis, patch repair of mitral annulus and valve, patch repair for left atrial dissection

## Abstract

**Introduction:**

Left atrial dissection is a rare event, typically associated with cardiac manipulation. We report the first case of a left atrial dissection caused by parasitic infectious endocarditis, which required the use of patch repair for the damaged mitral annulus and valve.

**Case Presentation:**

To treat heart failure in a 43-year-old man with left atrial dissection, we performed a patch repair of the mitral annulus and valve using autologous pericardium.

**Conclusion:**

We encourage novel surgery for complicated infectious endocarditis.

## Introduction

Left atrial dissection (LatD) is an extremely rare condition characterized by a false blood-filled cavity or lumen extending from the mitral annulus (MA) to the left atrium (LA) (1, 2). It is often linked to cardiac manipulation, myocardial infarction (MI) and blunt cardiac trauma (1, 2). Spontaneous LatD are associated with conditions such as cardiac amyloidosis, severe mitral annular calcification, and infectious endocarditis (IE) in some studies (1, 2). The majority of IE cases are caused by bacterial or fungal infestation (1–3). Here, we present a unique LatD case caused by parasitic infestation of heart, along with the first report using pericardial patch repair for posterior mitral annulus in a LatD associated with IE (1–4). To prevent reinfection, we utilized autologous pericardial patch to repair MA and mitral valve (MV).

## Case presentation

**Figure 1:**
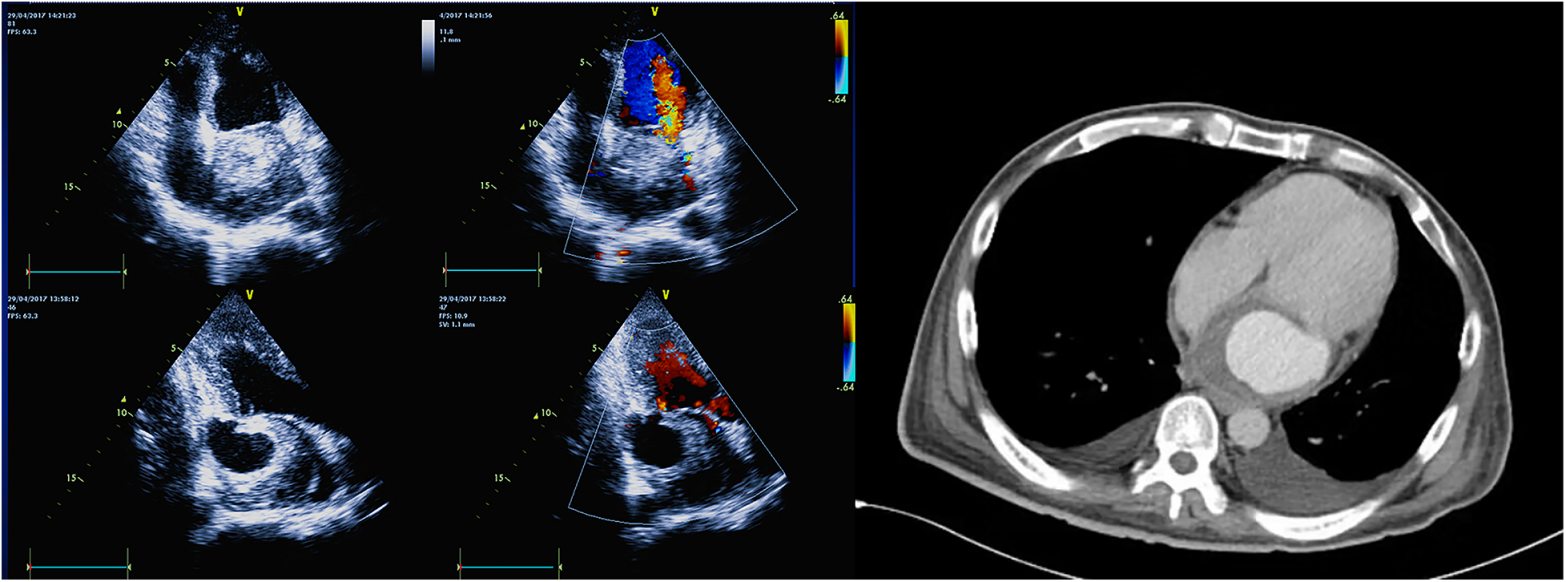
TTE showed a mass containing thrombus in the LA and a suspicious endocardial orifice beneath the root of the PML on the LV. CT revealed contrast filling the mass cavity.

A 43-year-old Chinese man with no prior history of cardiac surgery or trauma complained diarrhea, general fatigue, and lower extremities edema for three months. Laboratory test revealed an absolute eosinophil count of 5.69 cells × 10^9^/L with 37.8% eosinophils. Transthoracic echocardiogram (TTE) showed a 5.4 × 6.0 cm mass containing thrombus in the posterior wall of the left atrium (LA), causing functional mitral insufficiency and MV obstruction. In addition, there was a suspicious endocardial orifice beneath the root of the posterior mitral leaflet (PML) on the left ventricle (LV). Enhanced Computed tomography (CT) revealed the contrast filling the mass cavity, indicating intramural hematoma. (Figure 1) Left atrial dissection caused by parasitic infestation was suspected. Based on the patient's hemodynamic collapse, emergency surgery was necessary.

**Figure 2:**
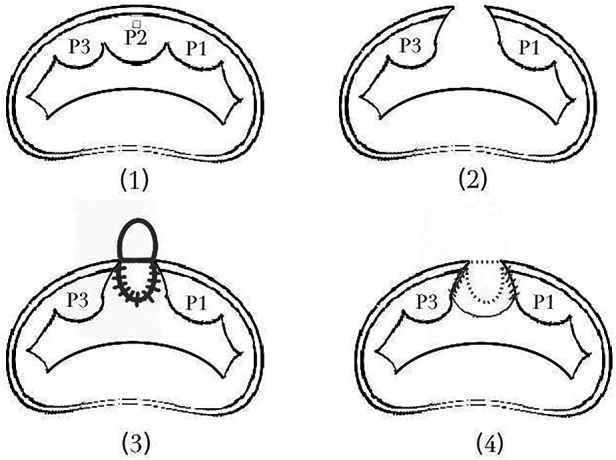
The procedure of patch repair for defect of LV, MV and MA. (2) To reach the orifice, we direct incised the P2, and cut through the posterior MA. (3) An ellipse-shape autologous pericardial patch was employed to reconstruct the defect of the LV and MA. (4) After reaching the proposed neo-annulus line, the rest of the ellipse was used to augment the PML.

The surgical procedures were recorded in Supplementary Material Video 1. By removing the thrombus and debris from the false cavity and excising the dissected endocardium, the surgeon found an orifice on the left ventricle beneath the root of the PML, which was confirmed as the entry of the LatD. Direct incision was performed in the P2, and the posterior MA was cut through to reach the orifice. An autologous pericardial patch with an ellipse shape was employed to reconstruct the defect of the LV and MA. After reaching the proposed neo-annulus line, the rest of the ellipse was used to augment the PML. An annuloplasty was performed with another pericardial band (refer to Figure 2). The reconstructed MV performed well under TEE after weaning from the cardiopulmonary bypass.

**Figure 3:**
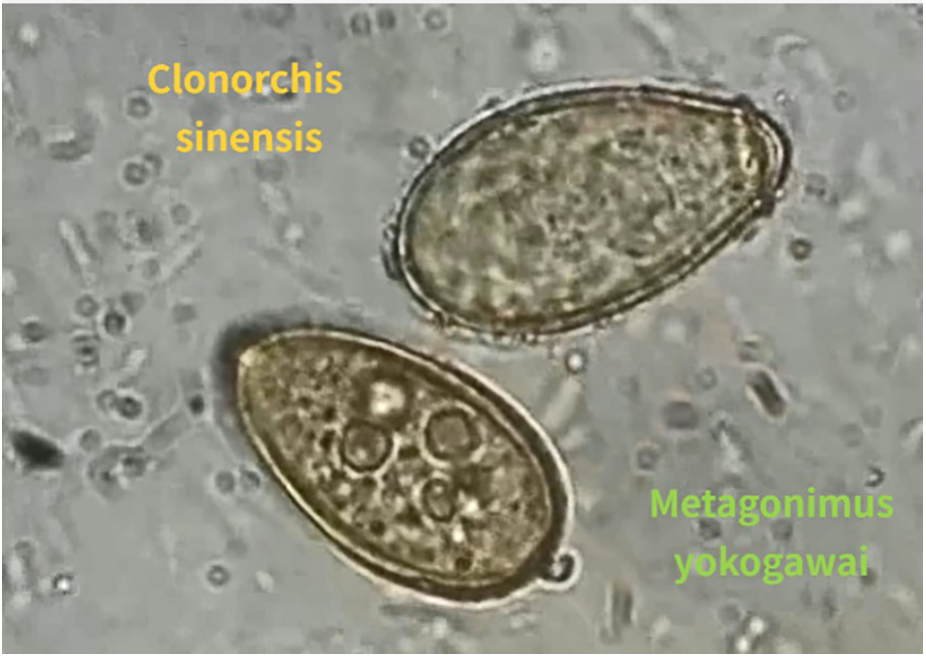
The ova of clonorchis sinensis (orange) and metagonimus yokogawai (green) in the stool examination.

Although the specimen produced no findings, the eosinophil count and the percentage of eosinophils were sharply decreased after surgery. The stool examination revealed the ova of Clonorchis Sinensis, Metagonimus yokogawai and Echinostoma (refer to Figure 3), confirming a LatD associated with parasitic infestation of heart. After completing anti-parasitic treatment, the patient was discharged and remained healthy during the 2-year follow-up period (5) (refer to Table 1).

**Table 1:** Timeline.

Time	Situation
3 Month Previously	Onset of diarrhea, general fatigue, and lower extremities edema
Initial Presentation	TTE showed a 5.4 × 6.0 cm mass in the posterior wall of the LA, causing functional mitral insufficiency and MV obstruction, and a suspicious endocardial orifice in the LV. Diagnosis of left atrial dissection was suspicious made. Hemodynamic instability required emergency surgery.
Surgery Day	Pericardial patch repair of mitral annulus and mitral valve was performed
2 weeks later	A stool examination revealed the oval of parasites. Anti-parasitic treatment was performed.
5 months later	Anti-parasitic treatment was finished. A stool examination showed negative.
2 years later	Patient remained healthy.

## Discussion

Left atrial dissection can result from various cardiac interventions, including both surgical procedures (primarily MV surgery) and catheter-based interventions (1, 2). Cases of LatD resulting from MI and cardiac trauma have also been recorded (1, 2). Fukuhara et al. reported only 7 cases of spontaneous LatD, one of which was related to IE without cardiac manipulation (1). With another case of LatD with IE in other report, both patients were caused by bacterial infection and died before emergency surgery could be performed (1–3). This might be the first report of LatD resulting from parasitic infestation of heart and survived after surgery.

Transesophageal echocardiography (TEE) is the preferred method for diagnosing LatD, but its efficacy and diagnostic value are still limited (1). Echocardiogram often shows a left atrial mass or a tamponade, particularly when there is thrombus attached (1). Visualizing the entry of the dissection or communication between the inflow and the LA can be challenging (1). A TTE was performed in the emergency room on our patient and revealed an ambiguous left atrial mass with regurgitation flow under the PML, which could have been misdiagnosed as a leaflet perforation. Enhanced CT imaging is also limited in identifying precisely when the thrombus filled in the false cavity.

Hemodynamic instability is mainly secondary to obstruction of MV inflow or pulmonary vein orifice and eventually becomes congestive heart failure and low-output syndrome, making conservative management impractical. Surgery was necessary for our patient (2).

To achieve successful repair, three main keys are the following: (a.) to gain adequate evacuation of the hematoma; (b.) obliteration of the false lumen; and (c.) addressing the entry point, if identified. If the communication is not repaired, the dissection may recur under persistent pressurized inflow (1, 2). Therefore, we debride thrombus and resect the dissected endocardium. To close the orifice in the LV, a posterior annulus cutting was necessary. However, the tissue of LA and MA is often delicate and should be preserved whenever possible. Encouraged by Masashi's successful application of the technique for patch repair of mitral annulus calcification, we proceeded with posterior MA cutting and patch repair (6). We chose the autologous pericardium as patch to prevent artificial graft reinfection.

## Conclusion

By introducing this rare parasitic infestation of LatD and its surgical details, we aim to encourage novel surgery for complicated infective endocarditis.

## Data Availability

The original contributions presented in the study are included in the article/**Supplementary Material**, further inquiries can be directed to the corresponding author/s.
