# Supplementary material for: Case Report: Pericardial patch repair of mitral annulus and mitral valve for a left atrial dissection caused by parasitic infective endocarditis
Source: Front Cardiovasc Med. 2023 Nov 28;10:1239019. doi: 10.3389/fcvm.2023.1239019 (PMC10715251; doi:10.3389/fcvm.2023.1239019)
Supplement: Supplementary file 1 [file Table1.docx]

**Timeline**

| Time | Situation |
| --- | --- |
| 3 Month Previously | Onset of diarrhea, general fatigue, and lower extremities edema |
| Initial Presentation | TTE showed a 5.4×6.0 cm mass in the posterior wall of the LA, causing functional mitral insufficiency and MV obstruction, and a suspicious endocardial orifice in the LV. Diagnosis of left atrial dissection was suspicious made. Hemodynamic instability required emergency surgery. |
| Surgery Day | Pericardial patch repair of mitral annulus and mitral valve was performed |
| 2 weeks later | A stool examination revealed the oval of parasites. Anti-parasitic treatment was performed. |
| 5 months later | Anti-parasitic treatment was finished. A stool examination showed negative. |
| 2 years later | Patient remained healthy. |

Table 1
